# Assessment of Isokinetics and Range of Motion of the Shoulder in Patients after Reverse Shoulder Arthroplasty in the Late Follow-Up Period

**DOI:** 10.3390/jcm12237351

**Published:** 2023-11-27

**Authors:** Katarzyna Ogrodzka-Ciechanowicz, Piotr Kurzeja, Tomasz Sorysz

**Affiliations:** 1 Institute of Clinical Rehabilitation, Faculty of Motor Rehabilitation, University of Physical Education, 31-571 Krakow, Poland; 2 Institute of Health Sciences, University of Applied Sciences in Nowy Targ, 34-400 Nowy Targ, Poland; piotrkurzeja@op.pl; 3 Trauma and Orthopedic Unit, Gabriel Narutowicz Municipal Specialist Hospital in Krakow, 31-215 Krakow, Poland; tsorysz@gmail.com

**Keywords:** reverse total shoulder arthroplasty, isokinetic test, range of motion

## Abstract

(1) Background: The aim of the study was to evaluate the peak torque (PT) in isokinetic conditions and the range of motion of the shoulder joint in patients after reverse total shoulder arthroplasty in the late treatment period. (2) Methods: The study included fifteen patients aged 60–70 years (13 women and 2 men). The comparison group consisted of 15 healthy subjects (12 women and 3 men) aged 60–69 years. The study included measurement of peak torque (PT) and the range of motion of the shoulder joint, assessed using the Biodex System 4 Pro set, and an electronic goniometer. We conducted tests at two different angular velocities (60°/s and 90°/s), taking into account the operated and non-operated limb and comparing the results to healthy subjects. The average time from surgery to functional examination was 16 months. (3) Results: The non-operated limb generated significantly higher PT values than the operated limb (*p* < 0.001). The healthy limb of patients from the comparison group generated significantly higher PT values than the operated limb of patients from the study group (*p* < 0.001). A significant improvement (*p* < 0.001) in the range of motion in the operated limb was achieved after rTSA. (4) Conclusions: In patients 18 months after the rTSA, the non-operated upper limb has significantly greater muscle strength in flexion/extension and abduction/adduction movements compared to the operated limb. The non-operated limb also has a significantly greater range of motion compared to the operated limb.

## 1. Introduction

Reverse total shoulder arthroplasty (rTSA) was developed in the 1980s as a treatment for rotator cuff arthropathy in older adults. Due to the effectiveness of the procedure, it began to be used as the treatment of choice for diseases and injuries of the shoulder joint involving damage to the rotator cuff [1]. Over the last few decades, shoulder arthroplasty has been constantly developing. Research indicates that total shoulder arthroplasty, both anatomic and reverse, is an effective treatment for shoulder pathology that provides significant pain relief and functional benefits [2].

Reverse total shoulder arthroplasty is used in multi-fragment fractures of the humeral head, in advanced degenerative disease or in chronic inflammation of rheumatological origin. However, the prerequisite for this treatment is complete damage to the rotator cuff. Complete damage to the tonic muscles of the shoulder joint does not allow for their regeneration, and the direct indication for surgery is persistent night pain [3,4,5,6].

Due to the change in joint anatomy and rotator cuff failure, there are also several differences in the physiotherapy process between an anatomical prosthesis and rTSA. After rTSA, muscle activation begins faster because there is no regeneration of any muscle tendons. Additionally, the activation of the phasic muscles of the shoulder joint is primarily directed at the deltoid muscle, including the following muscles: pectoralis major, trapezius, rhomboid, and serratus anterior. It is very important to be careful when working passively at the end range of motion—failure to centralize the endoprosthesis may damage the implant. It is also important that the safe range of motion is 120° of flexion, approximately 90° of abduction and a complete ban on passive extension, also in the late period of treatment. The range of movements for extension, adduction and internal rotation is functionally limited and reaches the range of being able to tuck the shirt into the pants behind the back. According to research, the safe movement sector for rTSA is the space between the sagittal plane and scapular plane of the shoulder joint [3,4,5,6].

Despite numerous limitations resulting from the structure of the prosthesis, patients point out that they can function without pain at rest, perform self-care activities without pain, undertake light household and professional work without pain and have the ability to sleep peacefully and safely [3,4,5,6].

However, the results of muscle strength assessment after rTSA vary depending on the testing method and observation period. According to Preuss et al., the literature on postoperative strength outcomes following rTSA is limited, and further studies in this area are warranted [7].

Muh et al. presented a two-year follow-up of 66 patients after rTSA. The authors found that rTSA as a reconstructive procedure improved function during short-term patient follow-up, but overall satisfaction was significantly lower in this patient population compared to patient populations previously reported in the literature [8]. Similar conclusions were reached by Frankle et al., who presented results for 60 patients with rTSA who were followed up for at least two years after the procedure. All patients were assessed pre- and postoperatively using the American Shoulder and Elbow Surgeons Score (ASES) pain and function scoring system and the visual analog scale (VAS) [9].

The literature does not provide clear information on how long it takes for patients to regain shoulder strength after rTSA. Sperling et al. states that, despite tests conducted at various times after the rTSA, it is not possible to determine the moment when strength functions return to normal or whether they return at all [10]. A lack of knowledge about the time it takes for patients to regain shoulder strength after rTSA limits our ability to confidently assess postoperative improvement and counsel patients regarding postoperative expectations. Additionally, there is a lack of research that would use objective research tools, e.g., Biodex System 4 Pro, and not only scales and questionnaires.

During the rehabilitation of patients with gait disabilities, the use of new technologies is becoming popular, e.g., mechatronic system for environmental rehabilitation of people with gait disabilities, which enable the restoration of the correct gait pattern [11]. It is therefore justified to try to evaluate and use such systems in patients with upper limb dysfunctions, which could contribute to improving the strength functions of the limb.

Due to the discrepancies in the assessment of joint function after rTSA, our own research undertook an isokinetic assessment of the shoulder joint.

The authors hypothesize that rTSA significantly improves the muscle strength of the operated shoulder joint.

The aim of the study was to evaluate the peak torque (PT) in isokinetic conditions and the range of motion of the shoulder joint in patients after reverse total shoulder arthroplasty in the late treatment period.

## 2. Materials and Methods

### 2.1. Study Design

This observational study was conducted in compliance with the Strengthening the Reporting of Observational Studies in Epidemiology (STROBE) statement: guidelines for reporting observational studies and the ethical standards of the Human Experimentation Committee of the institution in which the experiments were conducted or in accord with the Declaration of Helsinki of 1964 and its later amendments; it received approval from the Bioethics Committee [48/KBL/OIL/2022], and all patients gave their written informed consent [12].

The study included 18 patients of the Trauma and Orthopedic Department of the Gabriel Narutowicz Municipal Specialist Hospital in Kraków who underwent the rTSA procedure.

The indication for arthroplasty in all patients was degenerative changes in the shoulder joint. The average time from surgery to functional examination was 16 months (16.9 ± 2.28).

Inclusion criteria:

1. rTSA procedure performed due to degenerative joint disease and damage to the rotator cuff muscles;
2. No other diseases or injuries of the musculoskeletal system in the shoulder girdle;
3. No neurological diseases that could affect the course of the examination;

Exclusion criteria:

1. Inflammation and acute pain in the shoulder girdle;
2. Lack of written consent from the patient to participate in the study.

Immediately after the procedure, each patient had their upper limb immobilized in a brace for up to 4 weeks. Then, all patients underwent postoperative physiotherapy, which was carried out from the 4th to the 14th week after the procedure and included passive mobilization of the joint up to 90 degrees in the scapular plane, Codman’s pendulum exercises, isometric exercises of the deltoid muscle and gradual active mobilization of the shoulder joint, gradual strengthening of the joint muscles, reconstruction of the scapulohumeral rhythm and reconstruction of the range of motion in individual planes of movement, consistent with the structure of the endoprosthesis. The exercises were conducted 3 times a week. One session with a physiotherapist lasted one hour. Strengthening exercises were selected individually—the load was increased gradually with an increase in the range of motion and muscle control. The number of repetitions was 10–15; patients started with 1–2 series, ending in the last week with 4 series.

The comparison group consisted of 15 healthy subjects (12 women and 3 men), aged 60–69 (64.45 ± 3.12). This group consisted of healthy people who met the inclusion criteria (without injuries or diseases of the shoulder joint).

The research was conducted from January to November 2022 in the Functional Diagnostics Laboratory of the Central Scientific and Research Laboratory of the University of Physical Education in Krakow.

### 2.2. Procedures

The first measurement of ROM was performed before arthroplasty upon admission to the hospital, and the second measurement was performed 18 months after the rTSA, simultaneously with the assessment of muscle strength, which was performed once, 18 months after the procedure.

The study included measurement of peak torque (PT) in isokinetic conditions and the range of motion of the shoulder joint, assessed using the Biodex System 4 Pro set, and measurement of the range of motion using an electronic goniometer.

Conducting tests at two different angular velocities (60°/s and 90°/s), taking into account the operated and non-operated limb, and comparing the results to those obtained by healthy subjects enabled a reliable assessment of the biomechanical capabilities of the shoulder joint in people after rTSA.

### 2.3. Intervention

#### 2.3.1. Isokinetic Tests

The examination of muscle strength during isokinetic contraction of the shoulder joint included measurement during abduction (up to 90°) and adduction as well as flexion (up to 90°) and extension using the concentric-concentric protocol with angular velocities of 60°/s, 90°/s. The angular velocities were matched to the patients’ strength capabilities.

**Figure 1:**
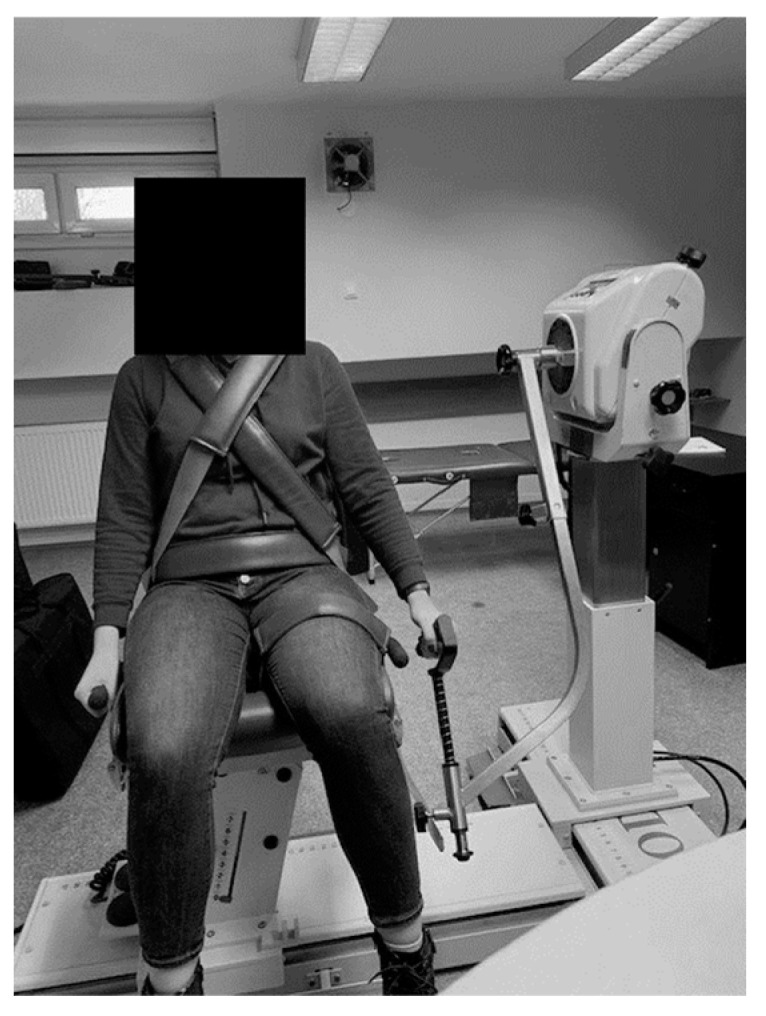
Configuration and position of the Biodex System 4 Pro for performing shoulder flexion/extension exercises (initial position).

A standard adapter for the upper limb was used to test the muscle strength of the shoulder joint. The equipment calibration procedure was carried out in accordance with the manufacturer’s recommendations. During the examination, the patient was in a sitting position, and the examined upper limb was straightened. In order to stabilize the body and eliminate compensatory movements, the patient was fastened to the chair with belts—on the chest and pelvis. The acromion process was taken as the axis of joint rotation. The subject then grasped the handle of the dynamometer. The initial position for each measurement was lowered arm along the torso (Figure 1).

The test protocol included two measurements for the abduction and adduction movements, separately for two angular velocities and the same for the flexion and extension movements. A single measurement lasted 1 min, during which the patient performed the maximum number of repetitions. Between subsequent series, in accordance with the isokinetic protocol, there was a 60 s rest break. Two repetitions, not counted for measurement, were allowed before each test so that the subject could get used to the position, speed and task [13,14].

During each attempt, peak torque (PT) [Nm/kg*%], i.e., peak torque per body weight, was assessed.

All patients participating in the study performed short “warm-up” exercises—free movements in the pain-free range in all possible planes of movement. After one-minute rest, participants performed the tests. Each test was preceded by entering information about the patient into the system and database and determining the dominant and operated limb. Then, after stabilizing the patient on the test chair, the measurement range of motion was set, the zero position of the limb was determined and the limb was weighed; then, the test was performed.

In each patient in the study group, the muscle strength of the operated and non-operated upper limb was assessed, and in the case of the healthy group, the strength of the dominant and non-dominant limb was assessed.

Each measurement included two series. For statistical purposes for our own research, the results of the peak value of the maximum torque of the greatest force for a given attempt were included in the analysis. This value indicates the maximum strength capabilities of the tested muscle group [15,16].

#### 2.3.2. Range of Motion

**Figure 2:**
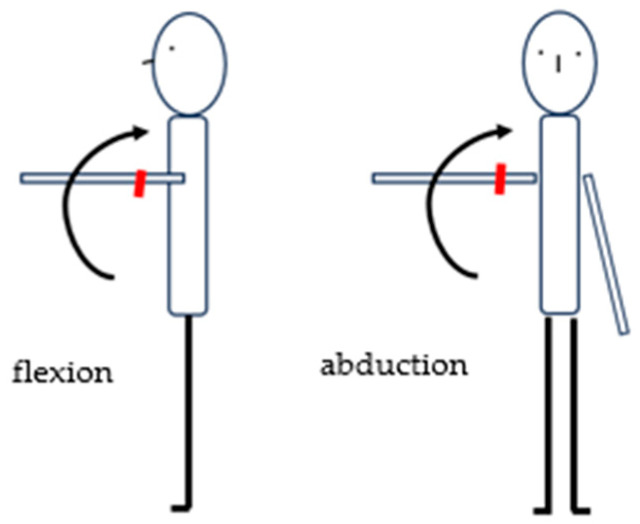
Measurement of the range of motion using K-FORCE Sens—location of the goniometer (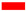).

Additionally, each patient had the range of motion of their shoulder joint measured using the K-FORCE Sens electronic goniometer. The measurement methodology was consistent with the recommendations proposed by Hayes et al. [17]. The test was performed in a standing position with the goniometer placed in the middle of the arm being tested (Figure 2).

#### 2.3.3. Outcome Measures

The measurement of muscle strength during isokinetic contraction was performed using the Biodex System 4 Pro station.

Biodex System 4 is a set used to assess and train neuromuscular muscles in the following conditions: isometric, isotonic (concentric and eccentric), isokinetic (eccentric and concentric), reactive eccentric and passive movement, with the possibility of fully archiving and exporting data for statistical analysis. The device is equipped with an electrically adjustable comfortable swivel chair that moves on a base and an electric dynamometer adjustable in 3 planes for performing tests/exercises on a diverse group of patients. A mobile workstation with a control panel is used to analyze the tests or exercises performed.

Biodex System 4 Pro (Biodex Medical System Inc., Shirley, NY, USA) is a multi-module, computerized dynamometer, commonly used in diagnostics and therapy in sports medicine, orthopedics, pediatric medicine, neurorehabilitation, geriatrics and scientific research. The dynamometer allows for the measurement of the maximum strength capabilities of various muscle groups. Isokinetic dynamometry is considered the gold standard in assessing muscle strength. The main goals of isokinetic testing are to determine muscle performance, track progress and test imbalances between body sides and agonist–antagonist muscle relationships. Reliability and repeatability of measurements is a key factor in this context [18].

The reliability of the force measurements was assessed using both older versions of Biodex and Biodex System 4 Pro. These tests showed moderate to excellent, mostly excellent, reliability at peak torques [19].

The K-FORCE Sens electronic goniometer (Kinvent™, Montpellier, France) is designed to assess, monitor and rehabilitate the range of motion. With the included application, it is possible to conduct therapy using feedback (biofeedback). The software allows you to test the range of motion in the joints and visualizes it on charts. Thanks to this, in addition to the diagnostic function, it also allows for conducting rehabilitation. It offers real-time biofeedback based on improvement in the range of motion over initial values, using inertial measurement sensors. According to the technical specification manual, measurement accuracy is 1°, and device deviation is 3°. Studies indicate the reliability and repeatability of the results of range-of-motion measurements using K-FORCE Sens [20].

### 2.4. Statistical Analysis

The analyses used the parametric *t*-test for independent variables and its non-parametric equivalent, the Mann–Whitney U test (when the distribution of variables was significantly different from the normal distribution). Paired *t*-test was used for repeated measurements. The level of significance was *p* = 0.05. The analyses were performed in the statistical environment R 3.6.3 and MS Office 2021 (16.0).

## 3. Results

Initially, 18 patients aged 60–70 were enrolled in the study. After meeting the inclusion criteria, 15 patients (13 women and 2 men) participated in the study, with a mean age of 66.37 ± 2.97. In all patients, the right limb was the dominant limb. The right shoulder joint was operated on in 13 patients, and the left shoulder joint was operated on in 2 patients. The comparison group consisted of 15 healthy subjects (12 women and 3 men) aged 60–69 years (64.45 ± 3.12). Patients agreed to participate in the study.

**Figure 3:**
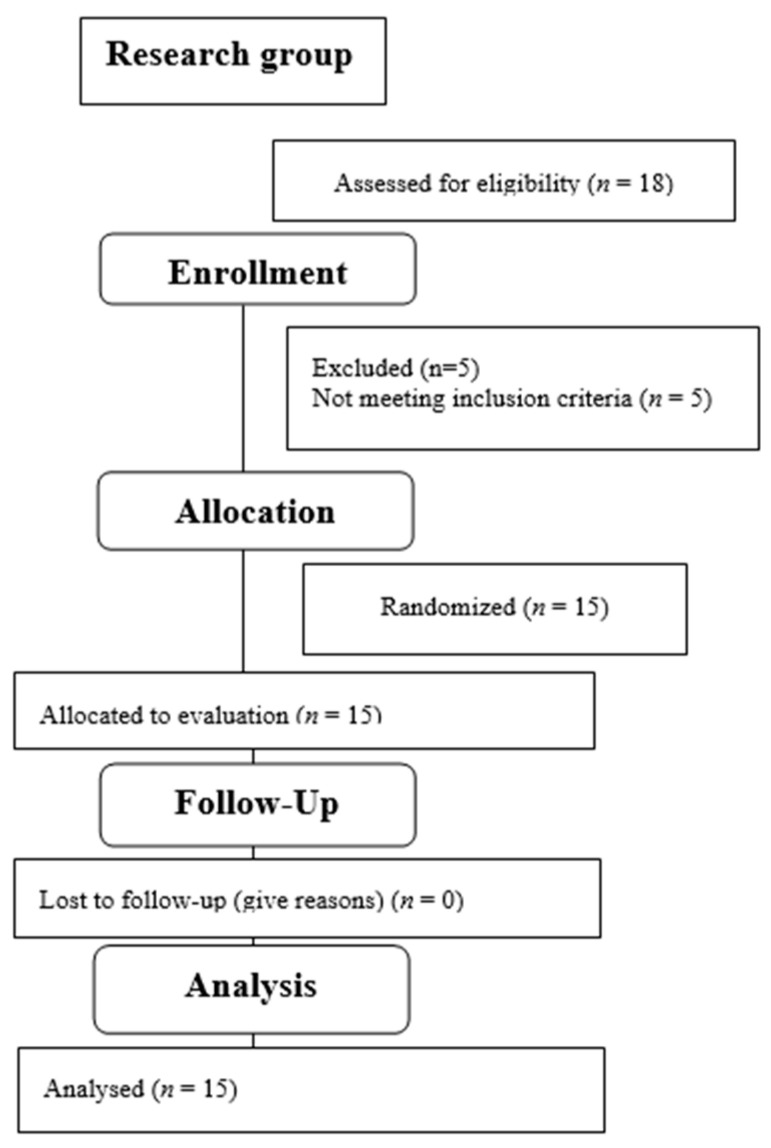
Flow diagram—the process of qualifying patients after rTSA for functional assessment.

Table 1 contains detailed anthropometric data for both groups. The qualification stage is presented in Figure 3.

The measurement values of peak torque (PT) were assessed in patients after rTSA and in the comparison group. The results were first compared between the operated side and the healthy side in the study group and then between the operated limb in the study group and the healthy limb in the healthy group.

The obtained results indicate statistically significant differences in the values of the PT index [Nm/kg*%] that occurred between the operated and non-operated upper limb of patients in the study group. The non-operated limb of patients from the study group generated significantly higher PT values than the operated limb (Table 2).

A comparison of the results between the study group and the healthy group also shows statistically significant differences in PT index values [Nm/kg*%]. The healthy limb of patients from the comparison group generated significantly higher PT values than the operated limb of patients from the study group (Table 3).

The measurement values of the range of motion in the shoulder joint among patients after rTSA were also assessed. The measurement results were first compared in the operated limb before arthroplasty and 18 months after rTSA, then between the operated and non-operated limbs in the study group.

Tests for adduction movement were not performed because this variable had a constant value of 0 in both measurements.

When comparing the range of motion of the operated limb before rTSA and 18 months after rTSA, all observed differences were statistically significant results. The average value of the shoulder flexion range of motion before rTSA was 40.37° (SD = 19.26°), while 18 months after rTSA, it was 97.59° (SD = 12.96°). The analysis showed that this is a statistically significant difference. Before rTSA, the median result of shoulder extension was 20.00° (min = 0°, max = 45°), while 18 months after rTSA, it was 35.00° (min = 10°, max = 50°). The analysis showed that the difference in the shoulder extension range of motion before rTSA and 18 months after rTSA is also a statistically significant result. Before rTSA, patients had an average abduction of 23.33° (SD = 15.63°), while 18 months after rTSA, it was already 95.19° (SD = 12.97°). The analysis showed that the difference in the abduction range of motion before rTSA and 18 months after rTSA is also statistically significant (Table 4).

When comparing the range of motion of the operated and non-operated upper limb before rTSA and 18 months after rTSA, 5 of the 6 observed differences were statistically significant results. The average value of flexion movement of the operated limb before rTSA was 40.37° (SD = 19.26°), while the average value of flexion movement of the non-operated limb during the same period was 160.59° (SD = 12.70°). This is a statistically significant difference. The average value of the flexion range of motion of the operated limb after rTSA was 97.59 (SD = 12.96°), and the same value in the case of the non-operated limb was 165.77° (SD = 7.47°). This difference is also a statistically significant result. The average extension of the operated limb before rTSA was 21.30° (SD = 11.47°), while the average extension of the non-operated limb before rTSA was 34.25° (SD = 8.73°). This is a statistically significant difference. One and a half years after rTSA, the average extension result for the operated limb was 32.41° (SD = 11.47°), and for the non-operated limb, it was 36.66° (SD = 6.79°). This difference is not a statistically significant result. Abduction of the operated limb before rTSA was on average 23.33° (SD = 15.63°), while the non-operated limb during this period, it achieved average results of 166.11° (SD = 6.97°). This difference is a statistically significant result. One and a half years after rTSA, the average result of abduction of the operated limb was 95.19° (SD = 12.97°), and of the non-operated limb, it was 166.29° (SD = 7.41°). This difference in values also constitutes a statistically significant result (Table 5).

## 4. Discussion

The aim of the study was to assess the muscle strength and range of motion of the shoulder joint in patients after reverse shoulder arthroplasty. The results obtained were compared with the results of healthy people of a similar age. The strength capabilities of the upper limb were assessed on the basis of isokinetic contraction, measured using Biodex System 4 Pro. The analysis showed that the operated limb of patients from the study group, both in terms of strength and range of motion, generates lower values of the assessed indicators in all of the above-mentioned comparisons. The only exception (it should be emphasized that, in this case, the operated limb also obtained a lower value than the non-operated limb, but the difference was statistically insignificant) is the extension of the shoulder joint assessed 18 months after the rTSA. The increase in the average value of the extension range of motion of the operated limb from the measurement before rTSA to the measurement 18 months after rTSA was 52%. For comparison, in the case of the flexion movement, the increase in value was 142%, and for the abduction movement, it was as much as 308%. The question arises: why did even a six times smaller percentage increase in the range of motion not result in a comparison and a statistically significant result? This is related to the reference range of motion values, i.e., the values of the non-operated, theoretically healthy limb. In the case of flexion and abduction movements, the non-operated limb after rTSA achieved values of 165.77° and 166.29°, which are results close to 170°, i.e., the norm for the range of these movements of the shoulder joint [21]. In contrast, the range of extension movement of the healthy, non-operated limb after the procedure is characterized by an approximately 29% deficit compared to the normal value of approximately 50° range of movement [21]. The extension of the shoulder joint is one of the first movements that become gradually limited with age, so given the age of the study group (60–70 years), this phenomenon can be explained by the deficit in movement of the non-operated limb [22]. All other results and comparisons showed that the operated limb in this group of patients, both before rTSA and 18 months after rTSA, has significantly lower values of muscle strength and range of motion, both in relation to the non-operated limb and in relation to the results of healthy people. Taking into account several factors, such as the complexity of the structure of the shoulder joint, the degree of difficulty and invasiveness of rTSA, the age of the patients and the time since the surgery, this is a result that could have been expected.

It is worth emphasizing that a statistically significant difference in results also occurs between the average range of motion of the operated limb before and after rTSA. The ranges of motion of extension, flexion and abduction increased by 52%, 142% and 308%, respectively. This proves how much progress the people in the study group have made in terms of their range of motion thanks to the reverse arthroplasty procedure and the 14-week post-operative rehabilitation program. However, considering all the results, it is easy to observe that people after this type of surgery, 18 months after the operation, still have clear deficits in muscle strength and range of motion in the shoulder joint of the operated limb.

The literature on related issues includes various opinions regarding the return of muscle strength and range of motion after rTSA. Ersen et al. also focused on an isokinetic assessment of muscle strength and range of motion in patients after rTSA [23]. The authors studied 41 patients with an average age of 70.8 years who underwent rTSA. The study used a CYBEX HUMAC 350 dynamometer (2009 version), and the comparison involved muscle strength and endurance assessed in abduction movements, external rotation and internal rotation. The most interesting result obtained by the authors, standing in some way in opposition to the result obtained in our own research, was observed in terms of muscle strength of the abduction movement. A statistical analysis did not show a significant difference in muscle strength in the abduction movement between the operated and non-operated limb. The average follow-up time for these studies was 34 months, which may be quite important in the context of the above-mentioned result of muscle strength in the abduction movement, as it indicates a longer average recovery time of patients after rTSA (time between the surgery and the dynamometer test) than in the case of our own research. In addition, the authors assessed the range of motion of flexion and abduction of the operated limb before and after rTSA. In this case, the values obtained yielded statistically significant results in the form of a smaller pre-treatment range of motion in flexion (on average 77.5° before rTSA and 111.6° the rTSA) and abduction (84.5° before rTSA and 108.8° after rTSA) of the operated limb.

Very interesting results were also obtained by Rienmüller et al., who assessed the shoulder joint in terms of range of motion and muscle strength among 13 patients 2 years after rTSA [24]. In addition, the authors also included radiological and electromyographic evaluation. All patients underwent postoperative rehabilitation lasting 3 months. The essence of the study was to compare the operated and non-operated limb. For range of motion, the study assessed abduction, flexion, external rotation and internal rotation. A statistical analysis showed a significantly smaller range of motion of the operated limb in each of the above-mentioned movements, which is consistent with the results obtained in our own research. However, the results for muscle strength were not so clear. Using the Biodex System 4 Pro apparatus, 6 movements were assessed: flexion, extension, abduction, adduction, external rotation and internal rotation. Only measurements of adduction movements, external rotation and internal rotation were statistically significant results. In each of these cases, the muscle strength on the operated side was significantly lower. The authors stated that the causes of this phenomenon were medialization of the center of rotation of the shoulder joint, dysfunction of the deltoid and teres minor muscles, elongation of the humerus and excessive co-activation of the antagonist muscles [24].

It is also worth mentioning important factors that may influence the postoperative results of the operated limb in patients after shoulder arthroplasty. Alsubheen et al. indicated that the muscle strength and range of motion results may be influenced by the preoperative range of motion and muscle strength (the lower the values before rTSA, the greater the improvement after rTSA), the age of the patients (the older the age, the less improvement in the results) and gender (the male gender showed greater improvement in muscle strength results) [3]. Moreover, Friedman et al. found that the postoperative results of the ASES questionnaire in this group of patients are also influenced by having no history of shoulder surgery, no history of tobacco use, smaller passive external rotation range of motion before rTSA and greater active external rotation range of motion before rTSA [25]. Regarding the range of motion itself, researchers found that a greater preoperative range of motion of the shoulder joint may result in a significantly greater range of motion after surgery. Of course, the presence or absence of post-procedure complications and different ASES results do not automatically imply changed indicators of muscle strength and range of motion, but it does create some awareness that, if a given factor affects the functionality of a limb, it may also disturb individual elements that are important components of this functionality.

A very important and often emphasized element of postoperative management in patients after rTSA is properly and regularly performed physiotherapy. Pereira et al., based on research conducted in 2022, observed that rTSA combined with rehabilitation procedures results in an upward trend in the range of motion of flexion, abduction and the combined movement of abduction with external rotation, and at the same time, it results in a downward trend in the range of combined movement of extension, adduction and internal rotation as well as in the strength of the deltoid muscle [26].

Edwards et al. compared two rehabilitation approaches after rTSA and noted that early and active rehabilitation was positively received by patients and may be more beneficial than a slower and more conservative approach, but there is no tangible evidence of an advantage of one type of treatment over the other. [27]. Bullock et al. emphasized that it is currently difficult to find a clear answer as to what type of rehabilitation procedure is best after rTSA and that this topic requires further research [6]. In our study, patients underwent standard rehabilitation, which is recommended for patients during hospital and post-hospital periods. Therefore, in the context of the results obtained, it becomes important to discuss the topic of modifying the rehabilitation plan to focus on regaining the proper proportions and strength and movement relationships of the joint after rTSA, taking into account the biomechanical capabilities of the prosthesis.

### Study Limitation

One element that would definitely be worth considering is to re-examine muscle strength and range of motion after a longer period of time (e.g., 3 years after the procedure), then compare the results obtained with those achieved so far. Another aspect that would also be worth addressing in the future is the comparison of different rehabilitation approaches and the effects they produce in terms of muscle strength and range of motion. Other elements whose impact would be worth assessing are patients’ BMI and tobacco use history. Interesting results could also be obtained by assigning patients to groups according to gender and comparing how the values of muscle strength and range of motion change in these groups. Focusing directly on muscle strength, an interesting alternative could be its assessment using another method, e.g., in isometric conditions. Moreover, it is also worth conducting tests at different times of the day, as scientific reports indicate that the generated muscle strength may change depending on the time of day [28].

Research on patients after rTSA yields many interesting and sometimes surprising results. Some discrepancies in the results are visible, and researchers themselves often emphasize that this topic requires better understanding and analysis. This creates very good prospects for conducting further research in this group of patients and for future observations as a direct continuation of our own research.

The total shoulder arthroplasty, including rTSA, is an increasingly popular method of treating irreversible changes in the shoulder joint. Due to the growing interest in this method and the ever-increasing number of procedures performed, scientific analysis and assessment of all aspects of this field or factors related to it are key to improving postoperative effects and results and achieving the highest possible patient satisfaction. Deepening the knowledge of this method, as well as discovering new relationships, refining existing techniques and constantly analyzing and looking for areas for improvement, is extremely important for medicine and physiotherapy as well as their future development. Critical thinking and research into issues in this field actively contributes to improving the functional capabilities and quality of life of patients. This improvement should always be the primary goal and objective of every doctor and physiotherapist.

## 5. Conclusions

In patients 18 months after the rTSA procedure, when assessed in isokinetic conditions, the non-operated upper limb has significantly greater muscle strength in flexion/extension and abduction/adduction movements compared to the operated limb. Furthermore, the non-operated limb also has a significantly greater range of motion compared to the operated limb. The difference in the range of motion (with the exception of extension movement) is clear both before rTSA and 18 months after rTSA.

When assessed in isokinetic conditions, the muscle strength of the shoulder joint in flexion/extension and abduction/adduction movements with angular velocities of 60°/s, 90°/s, generated by healthy subjects, is significantly and clearly higher than that obtained for the operated limbs of people after rTSA.

## Figures and Tables

**Table 1:** Anthropometric data of the subjects in both groups.

Variable	Research x ± SD; (min/max)	Healthy x ± SD; (min/max)
Age [years]	66.37 ± 2.97 (60/70)	64.45 ± 3.12 (60/69)
Height [cm]	165.81 ± 5.71 (158/179)	163.45 ± 4.37 (159/181)
Body weight [kg]	76.11 ± 11.62 (59/108)	73.20 ± 9.76 (61/101)

**Table 2:** Comparison of the results of peak torque measurements of the operated and non-operated limb in the study group.

M	V [°/s]	Side	*PT* [Nm/kg*%]			*t*/*U*	*p*
			x ± Std	Me	min–max		
Abd	60	Op		22.60	12.00–36.00	106.50 U	<0.001 *
		N-op		31.80	20.50–51.40		
	90	Op		24.70	14.10–41.30	55.00 U	<0.001 *
		N-op		36.10	29.20–55.90		
Add	60	Op	13.56 ± 3.46			−6.76 t	<0.001 *
		N-op	23.07 ± 6.44				
	90	Op		12.70	5.70–21.30	116.00 U	<0.001 *
		N-op		21.70	10.70–44.40		
Flex	60	Op	24.89 ± 7.94			−3.84 t	<0.001 *
		N-op	33.77 ± 9.04				
	90	Op	29.76 ± 8.42			−4.23 t	<0.001 *
		N-op	41.01 ± 10.95				
Ext	60	Op		16.60	10.20–37.20	119.00 U	<0.001 *
		N-op		25.90	14.30–46.50		
	90	Op	22.25 ± 7.14			−3.47 t	0.001 *
		N-op	29.82 ± 8.79				

PT—peak torque; t/U—test statistics; V—angular velocity; *p*—statistical significance; x—mean; Std—standard deviation; Me—median; min—minimal score; max—maximum score, Add—adduction; Abd—abduction; Flex—flexion; Ext—extension; M—move; Op—operated side, N-op—non-operated side; *—statistically significant.

**Table 3:** Comparison of the results of peak torque measurements of the operated limb in the study group with the results of the healthy group.

M	V [°/s]	Group	PT [Nm/kg*%]			*t*/*U*	*p*
			x ± SD	Me	min–max		
Abd	60	rTSA		22.60	12.00–36.00	0.00 U	<0.001 *
		H		68.90	37.30–99.50		
	90	rTSA		14.10	41.30–24.70	0.00 U	<0.001 *
		H		55.60	103.90–79.70		
Add	60	rTSA		13.20	5.20–19.60	0.00 U	<0.001 *
		H		67.80	41.30–102.60		
	90	rTSA		12.70	5.70–21.30	0.00 U	<0.001 *
		H		81.50	48.60–105.30		
Flex	60	rTSA		26.10	10.60–44.40	0.00 U	<0.001 *
		H		74.30	45.40–100.60		
	90	rTSA	29.76 ± 8.42			−17.56 t	<0.001 *
		H	87.66 ± 14.92				
Ext	60	rTSA		16.60	10.20–37.20	0.00 U	<0.001 *
		H		66.90	41.50–120.20		
	90	rTSA		21.20	10.60–39.30	0.00 U	<0.001 *
		H		75.10	54.10–125.80		

rTSA—research group; H—upper extremity in healthy group; *—statistically significant.

**Table 4:** Results of measuring the range of motion in the operated limb before rTSA and 18 months after rTSA.

M	Measurement	ROM			*t*/*W*	*p*
		x ± SD	Me	min–max		
Flex	Before	40.37 ± 19.26			−12.92	<0.001 *
	After	97.59 ± 12.96				
Ext	Before		20.00	0–45	0	<0.001 *
	After		35.00	10–50		
Abd	Before	23.33 ± 15.63			−21.34	<0.001 *
	After	95.19 ± 12.97				

ROM—range of motion; Before—before rTSA; After—after rTSA; *—statistically significant.

**Table 5:** Results of the range of motion measurement in the operated and non-operated limb before rTSA and 18 months after rTSA.

M	Mesurement	Op	N-Op	*p*
		x ± SD	x ± SD	
Flex	Before	40.37 ± 19.26	160.59 ± 12.70	0.0113 *
	After	97.59 ± 12.96	165.77 ± 7.47	0.017 *
	*p*	<0.001 *	0.348	
Ext	Before	21.30 ± 13.49	34.25 ± 8.73	<0.001 *
	After	32.41 ± 11.47	36.66 ± 6.79	0.534
	*p*	<0.001 *	0.254	
Abd	Before	23.33 ± 15.63	166.11 ± 6.97	0.039 *
	After	95.19 ± 12.97	166.29 ± 7.41	<0.001 *
	*p*	<0.001 *	0.386	

*—statistically significant.

## Data Availability

The minimal data set is contained within our paper.
